# Farnesoid X Receptor Regulated Sepsis‐Induced Abnormal Bile Acid Metabolism via the Fibroblast Growth Factor 15/Fibroblast Growth Factor Receptor 4 Pathway

**DOI:** 10.1002/iid3.70155

**Published:** 2025-04-07

**Authors:** Ziyang Zhou, Dan Xu, Liou Huang, Yuhui Cui, Hui Chen, Jianguo Tang

**Affiliations:** ^1^ Trauma‐Emergency & Critical Care Medicine Center Shanghai Fifth People's Hospital Affiliated to Fudan University Shanghai China; ^2^ Joint Center for Translational Medicine, Shanghai Fifth People's Hospital, Fudan University and School of Life Science East China Normal University Shanghai China

**Keywords:** bile acid, farnesoid X receptor, FGF15/FXFR pathway, metabolomics, sepsis

## Abstract

**Objective:**

The study aims to investigate the mechanism of Farnesoid X receptor (FXR) activation in sepsis‐induced abnormal bile acid metabolism and the metabolism status of each bile acid type.

**Methods:**

The sepsis mouse model was developed via lipopolysaccharide intraperitoneal injection and confirmed via hematoxylin and eosin (H&E) staining. FXR agonist activated the FXR/fibroblast growth factor (FGF)15/FGFR pathway via quantitative real‐time polymerase chain reaction and Western blot. Consequently, metabolomics and bioinformatics analysis were conducted to identify the alterations in each kind of bile acid content following FXR agonist/inhibitor intervention.

**Results:**

The H&E staining indicated that FXR activation alleviates the liver injury of the sepsis mouse model. The increased FGF15 and FXFR expression levels and decreased CYP7A1 demonstrated FXR/FGF15/FGFR pathway activation following FXR agonist treatment. Furthermore, total bile acid, interleukin (IL)‐6, and tumor necrosis factor‐α concentrations were downregulated after FXR activation, whereas IL‐10 concentration was upregulated, indicating the alleviated effect of FXR agonist in sepsis. Consequently, metabolomics and bioinformatics analysis determined that T‐a‐MCA were downregulated in both FXR agonist and inhibitor groups, whereas six bile acid types were altered in the control group.

**Conclusion:**

FXR activation was crucial in alleviating sepsis‐induced hepatic injury and cholestasis through the FGF15/FGFR signaling pathway, and FXR may act as a potential preventive and intervention target of sepsis.

## Graphical abstract

Farnesoid X receptor regulated sepsis‐induced abnormal bile acid metabolism via the FGF15/FGFR4 pathway.

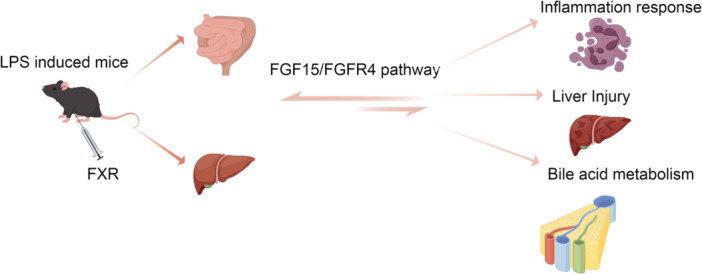

## 1 Introduction

Sepsis is, a life‐threatening organ dysfunction caused by a dysregulated host response to infection and encompasses diverse complications [1, 2]. Among them, refractory dysregulation of bile acid metabolism is generally acknowledged as one of the severe complications of sepsis, which causes cholestasis and seriously threatens the prognosis of patients with sepsis. Bile acid biotransformation has been impaired within hours after sepsis occurrence, causing cholestasis, which manifests as an early complication compared to other liver function impairments [3]. Thus, plasma bile acid levels demonstrate a significant potential as a diagnostic and prognostic marker for sepsis.

Nowadays, bile acid has become increasingly known as a pivotal signaling factor and metabolism‐regulating factor [4], and numerous pathological processes have been confirmed to involve bile acid metabolism misregulation [5]. Interestingly, most of the bile acid metabolism‐related diseases are crucial to farnesoid receptor (FXR), a member of the nuclear receptor superfamily that predominantly localizes in the human liver and intestine, thereby exerting crucial regulatory roles in maintaining bile acid homeostasis as well as lipid and glucose metabolism. As early as a decade ago, FXR was inseparable from obesity and insulin resistance [6, 7]. Afterward, cholestasis [8], nonalcoholic fatty liver disease (NAFLD) and nonalcoholic steatohepatitis (NASH) [9, 10, 11], alcohol‐associated liver disease [12, 13], and drug‐induced liver injury [14] were all related to FXR irregulation. FXR activation via obeticholic acid in the mice with acute liver injury was confirmed to alleviate lipopolysaccharide (LPS)‐induced liver injury. Furthermore, the anti‐inflammatory effects of FXR were illustrated [15], indicating the potential role of FXR activation in sepsis alleviation. Recent investigations on bile acid metabolism and FXR abnormalities revealed that bile acids were a subset of danger‐associated molecular patterns, which activated the NLRP3 inflammasome signals 1 (Inflammasome Priming) and 2 (Inflammasome Activation) in macrophages, and FXR negatively regulate inflammasomes in bile acid metabolism reducing [16, 17]. However, the classic pathway of bile acid metabolism, FXR/fibroblast growth factor (FGF)15/FGFR pathway, remains unknown regarding sepsis. Thus, our work focuses on this classic pathway and attempts to determine the underlying mechanism of FXR regulation in sepsis‐induced cholestasis via this classic pathway.

Fibroblast growth factor 15 (FGF15), orthologous to human FGF19, whose encoded protein plays a crucial role in bile acid negative regulation, neural crest cell migration, and bacterium response. FGF15 protein in bile acid metabolism acts as a kind of ligand to bind the fibroblast growth factor receptor 4 (FGFR4) and together constitutes a bile acid negative feedback pathway in the hepatoenteric cycle. Excessive bile acid acts as an activating molecule to activate the FXR throughout the regulatory procedure, and then the FGF15 gene transcription is regulated, which improves FGF15 protein secretion [18]. Subsequently, FGF15 enters the liver and binds with FGFR through the hepatoenteric cycle to inhibit hepatic CYP7A1 gene expression. The CYP7A1 gene encoded production is the rate‐limiting enzyme that is involved in the classical pathway of bile acid synthesis, which reduces bile acid production [19, 20, 21]. Therefore, we infer that the FXR/FGF15/FGFR pathway may serve as a pivotal mechanism in the sepsis‐induced abnormal bile acid metabolism in bile acid metabolism disorder.

The current study revealed that hepatic injury and cholestasis alleviation were attributed to the FXR activation through the FXR/FGF15/FGFR pathway. Bile acid metabolism and inflammatory responses demonstrated improved trends upon FXR agonist treatment. Furthermore, metabolomics testing and analysis were conducted to perform a deeper exploration of 41 kinds of bile acid metabolisms and the results indicated several bile acid metabolism alternations. The results we obtained indicated that FXR may serve as a potential target in the preventive intervention for sepsis, and FXR agonist may become a potential preventive option for sepsis‐induced cholestasis.

## 2 Materials and Methods

### 2.1 Animals

A total of 24 male C57 mice, aged between 6 and 8 weeks and weighting 20–25 g, were procured from Shanghai Slac Laboratory Animal Co. Ltd. (Shanghai, China). The mice were randomly allocated into four groups (control, LPS model, LPS + FXR agonist, and LPS + FXR inhibitor), consisting of six mice in each group. All the mice were housed in an environment with adequate food and water, a constant temperature of 25°C, and a light‐dark cycle alternating every 12 h. Mice in the model, the FXR agonist, and the FXR inhibitor groups received LPS (from *Escherichia coli* 055:B5, L8880, Solarbio, China) injections (10 mg/mL/kg), whereas those in the FXR agonist group and the FXR inhibitor group received intraperitoneal GW4064 (MCE, USA) (30 mg/kg) 5 days before the LPS injection and the intraperitoneal GUGGULSTERONE (MCE, USA) (100 mg/kg) 7 days before the LPS injections, respectively. Mice in the control group received only PBS injections with equal volume. The condition of each mouse was observed for 24 h after the construction of the animal model until the extraction of experimental materials from the mice. Specifically, after all the mice were anesthetized with potassium chloride solution (5%, 0.2 mL), their eyeballs were extracted to collect serum samples then the mice were euthanatized by instantaneous cervical dislocation and their livers and ileum were dissected for further research. The present study received approval from the Institutional Animal Care and Use Committee of the East China Normal University (m20240810).

### 2.2 Hematoxylin and Eosin Staining

The paraffin‐embedded mice liver samples were sliced into sections, then deparaffined, hydrated, and subsequently stained with H&E (Sigma, USA). An optical microscope (Olympus Optical Co. Ltd., Tokyo, Japan) was used for visualizing histopathological changes in mice livers at a ×200 magnification.

### 2.3 Quantitative Real‐Time Polymerase Chain Reaction

Total RNA was extracted from the livers and ileum tissue with the RNAios plus (TAKARA, Japan), and cDNA was then synthesized with the cDNA Synthesis Kit (Cwbio, China). Subsequently, the qRT‐PCR reaction system was constructed with SYBR®Premix Ex Taq (Mxbio, China), and the reaction was conducted with reaction conditions as 1 cycle of 95°C for 5 min, 40 cycles of 95°C for 15 s, and 60°C for 30 s, and 1 cycle of 60°C for 2 min. The mRNA relative expression levels of FGF15, FGFR, and CYP7A1 were calculated using the $2^{-\Delta \Delta C_{T}}$ method with GAPDH as a reference for normalization. Supporting Information S2: Table 1 presents all primer sequences.

### 2.4 Western Blot

The livers and ileum tissue of mice were sliced into small pieces and ground in radioimmunoprecipitation assay lysis buffer (Beyotime, China) with protease and phosphatase inhibitors (Roche, Swiss) for total protein isolation. Protein was quantified with the BCA Protein Assay Kit (Beyotime, China) and added into each well of the SDS‐PAGE gel at a 20‐μg mass for electrophoresis. Afterward, the protein in the gel was immediately transferred onto the polyvinylidene fluoride membrane and blocked with skim milk. Subsequently, the membrane was incubated with the primary antibodies, including anti‐GAPDH mouse monoclonal antibody (BBI, China, 1:5000), anti‐FGF‐15 rabbit polyclonal antibody (Abcam, UK, 1:1000), anti‐FGFR mouse monoclonal antibody (Proteintech, China, 1:1000), anti‐cytochrome P450 7A1 rabbit polyclonal antibody (ZENBIO, China, 1:1000), anti‐BESP (Wuhan Sanying, China, 3C11D5, 1:1000) and anti‐ABCC2 (Affinity, US, DF3873, 1:1000) at 4°C overnight. Secondary antibodies, including HRP‐conjugated goat antimouse IgG and HRP‐conjugated goat antirabbit IgG (BBI, China, 1:5000) were used to incubate the membrane at room temperature for 2 h after membrane washing. The protein bands on the membrane were visualized and imaged with the ECL luminescence method in the chemiluminescence imaging apparatus (4600, Tanon, Shanghai, China).

### 2.5 Automated Blood Biochemical Analysis

The total bile acid concentration in the serum of mice was detected with the Total Bile Acids Quantification Kit (Clinisciences, China) in a full‐automatic biochemical analyzer (HF240, HKANGYU, China).

### 2.6 Enzyme‐Linked Immunosorbent Assay (ELISA)

Interleukin (IL)‐6, tumor necrosis factor (TNF)‐α, and IL‐10 concentrations in the serum of mice were detected with mouse IL‐6 Elisa Kit, mouse TNF‐α Elisa Kit, and mouse IL‐10 Elisa Kit (MEXN, China), respectively. The Elisa experiment was performed following the instructions of kits and standard protocol. The OD value was recorded and analyzed to calculate the content of the three inflammatory factors.

### 2.7 Metabolomics Analysis

The standard solutions of 41 bile acids (Supporting Information S3: Table 2) and the internal standard working solutions of CA‐d4 and GCA‐d5 were prepared with corresponding standards and methanol. Subsequently, the serum samples that underwent homogenization, centrifugation, liquid–liquid extraction, vacuum drying, and redissolving were dissolved into A‐phase (0.01% formic acid aqueous solution) and B‐phase (acetonitrile) mixtures and separated with CORTECS UPLC C18 (100 × 2.1 mm, 1.6 μm) (Waters, USA). Supporting Information S4: Table 3 presents the mobile phase gradient setting. The mass spectrometry (MS) detection after liquid chromatography (LC) separation was completed with SCIEX Triple Quad™ 6500+ (SCIEX, USA) under the mode of ESI ion source, negative MRM ion. Supporting Information S5: Table 4 shows the MRM ion pair of the target and corresponding MS parameters. The standard curves were developed utilizing the data of working standard solutions analyses. Samples were assessed in the combinations of blank and standard samples with the recovery rates for bile acids identified based on the peak area to ensure the quality of analyses. Eventually, target bile acid concentrations in samples were identified with the internal standard method.

### 2.8 Metabolomics Data Analysis

The data was imported into the SIMCA‐P software package for further analysis. The principal component analysis (PCA) was first conducted after data normalizing to assess the separability among four groups. Fold change (FC) analysis and *t*‐test were used to conduct differential analysis, with results visualized via volcano plots. Subsequently, we conducted a hierarchical clustering analysis according to the significant differential metabolites within each group to evaluate the rationality of candidate differential bile acids, and the results were visualized using heatmaps. The box plot of each bile acid was plotted to reveal the bile acid contents and discrepancies with improved clarity.

### 2.9 Statistical Analysis

GraphPad Prism (8.0.1) was used for all the statistical analysis and relative chart drawing. All the data was presented as mean ± standard deviation, which was calculated with a one‐way analysis of variance or student's *t*‐test. *p *< 0.05 indicated statistical significance.

## 3 Results

### 3.1 FXR Activation Improved Sepsis‐Induced Liver Injury

**Figure 1:**
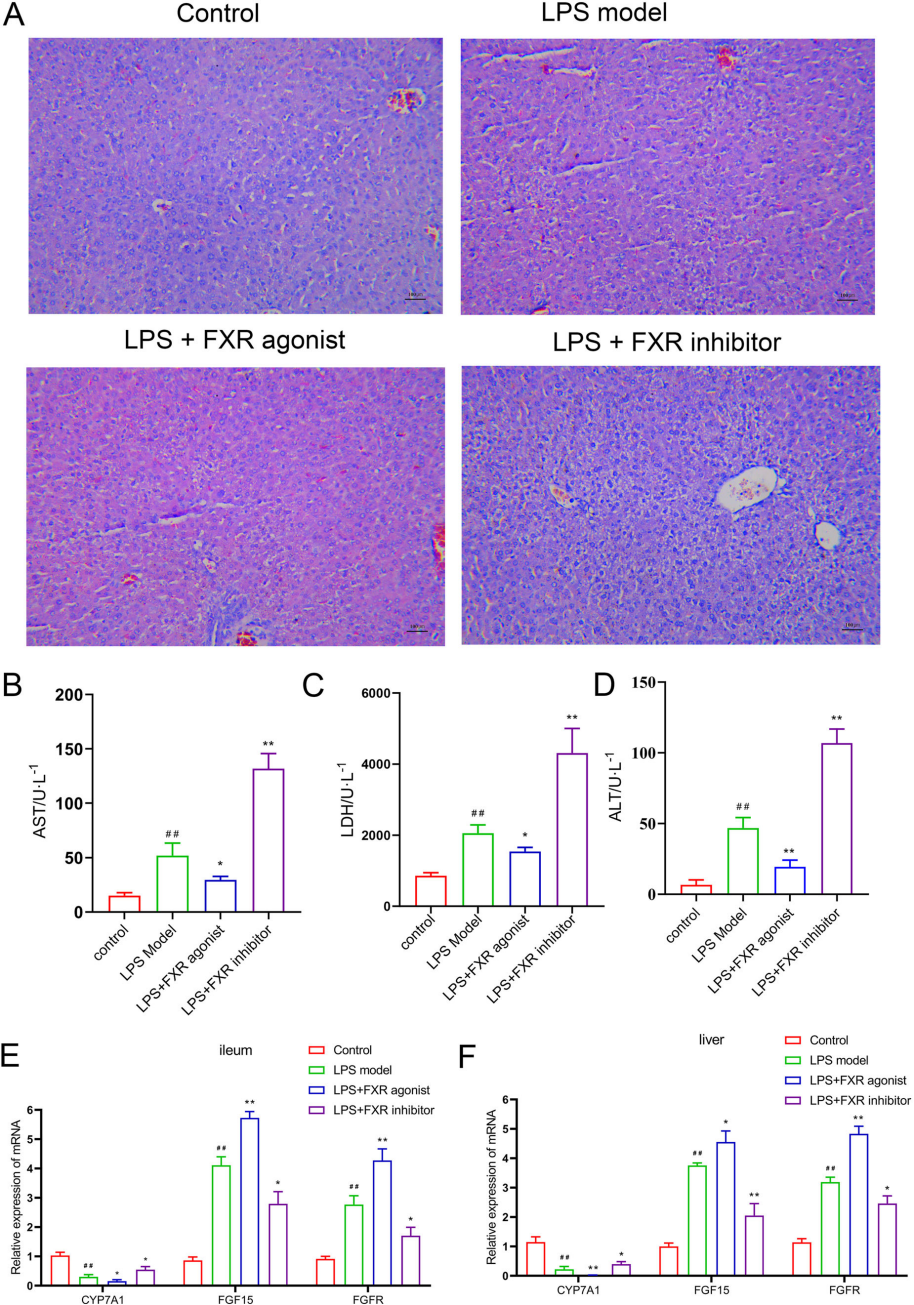
Association of FXR activation with sepsis‐induced hepatic injury and the expression levels of the relative genes in the FGF15/FGFR4 pathway. (A) H&E staining of mouse liver tissue from the control, LPS model, LPS + agonist, and LPS + inhibitor groups (scale bar indicates 100 μm). (B) The level of AST in liver of sepesis‐induced mice. (C) The level of LDH in liver tissues. (D) The ALT level in liver tissues. (E) The expression level of FGF15, FGFR, and CYP7A1 gene expressions in the ileum tissue. (F) FGF15, FGFR, and CYP7A1 gene expressions in the liver tissue. ^##^ *p* < 0.01, control versus LPS model; *p < 0.05, **p < 0.01, LPS model versus LPS + FXR agonist/inhibitor. FXR, farnesoid X receptor; H&E, hematoxylin and eosin; LPS, lipopolysaccharide.

The mouse model of sepsis was constructed via LPS to investigate the biological effect of the FXR in sepsis and its mechanism. Behavioral observation results indicated that the mice in the FXR inhibitor group demonstrated the worst physiological conditions, encompassing depression, abnormal eating and excretion, and dull fur. Conversely, the mice in the FXR agonist group showed better physiological conditions than LPS models. The specific behaviors and states of the mice are detailed in Table 1. H&E staining results of liver tissue revealed that FXR activation alleviated the arrangement disarray and hepatocyte vacuolization, whereas FXR inhibition aggravated the aforementioned liver injury conditions compared with the LPS model (Figure 1A). Also, liver damage indexes including AST, LDH, and ALT was examined for further evaluating liver damage, suggesting that FXR agnist restored liver damage induced by LPS (*p* < 0.05, *p *< 0.01, Figure 1B–D).

**Table 1:** The behaviors and physiological conditions of mice.

Mouse number and group	Description of mouse behaviors and states
Control 1	Vigorous physical activity, consistent dietary and hydration practices, regular patterns of urination and defecation, smooth and lustrous coat, prompt responsiveness, and robust resistance to external stimuli
Control 2	Vigorous physical activity, consistent dietary and hydration practices, regular patterns of urination and defecation, smooth and lustrous coat, prompt responsiveness, and robust resistance to external stimuli
Control 3	Vigorous physical activity, consistent dietary and hydration practices, regular patterns of urination and defecation, smooth and lustrous coat, prompt responsiveness, and robust resistance to external stimuli
Control 4	Vigorous physical activity, consistent dietary and hydration practices, regular patterns of urination and defecation, smooth and lustrous coat, prompt responsiveness, and robust resistance to external stimuli
Control 5	Vigorous physical activity, consistent dietary and hydration practices, regular patterns of urination and defecation, smooth and lustrous coat, prompt responsiveness, and robust resistance to external stimuli
Control 6	Vigorous physical activity, consistent dietary and hydration practices, regular patterns of urination and defecation, smooth and lustrous coat, prompt responsiveness, and robust resistance to external stimuli
LPS 1	Depression, disinterest in physical activity, diminished appetite and fluid intake, watery stools, decreased urine output, dull and unkempt coat, sluggish response to external stimuli, weakened ability to withstand external stressors
LPS 2	Depression, disinterest in physical activity, diminished appetite and fluid intake, watery stools, decreased urine output, dull and unkempt coat, sluggish response to external stimuli, weakened ability to withstand external stressors
LPS 3	Depression, disinterest in physical activity, diminished appetite and fluid intake, watery stools, decreased urine output, dull and unkempt coat, sluggish response to external stimuli, weakened ability to withstand external stressors
LPS 4	Depression, disinterest in physical activity, diminished appetite and fluid intake, watery stools, decreased urine output, dull and unkempt coat, sluggish response to external stimuli, weakened ability to withstand external stressors
LPS 5	Depression, disinterest in physical activity, diminished appetite and fluid intake, watery stools, decreased urine output, dull and unkempt coat, sluggish response to external stimuli, weakened ability to withstand external stressors
LPS 6	Depression, disinterest in physical activity, diminished appetite and fluid intake, watery stools, decreased urine output, dull and unkempt coat, sluggish response to external stimuli, weakened ability to withstand external stressors
LPS+FXR agonist 1	Slight depression, slight decrease in activity, minor reduction in appetite and fluid intake, relatively softer stools, marginally reduced urine output, dull coat, delayed response to external stimuli, slightly weakened resistance to external stress factors
LPS+FXR agonist 2	Slight depression, slight decrease in activity, minor reduction in appetite and fluid intake, relatively softer stools, marginally reduced urine output, dull coat, delayed response to external stimuli, slightly weakened resistance to external stress factors
LPS+FXR agonist 3	Slight depression, slight decrease in activity, minor reduction in appetite and fluid intake, relatively softer stools, marginally reduced urine output, dull coat, delayed response to external stimuli, slightly weakened resistance to external stress factors
LPS+FXR agonist 4	Slight depression, slight decrease in activity, minor reduction in appetite and fluid intake, relatively softer stools, marginally reduced urine output, dull coat, delayed response to external stimuli, slightly weakened resistance to external stress factors
LPS+FXR agonist 5	Slight depression, slight decrease in activity, minor reduction in appetite and fluid intake, relatively softer stools, marginally reduced urine output, dull coat, delayed response to external stimuli, slightly weakened resistance to external stress factors
LPS+FXR agonist 6	Slight depression, slight decrease in activity, minor reduction in appetite and fluid intake, relatively softer stools, marginally reduced urine output, dull coat, delayed response to external stimuli, slightly weakened resistance to external stress factors
LPS+FXR inhibitor 1	Severe depression, minimal activity, severely diminished appetite and fluid intake, watery stools, significantly reduced urine output, dull and unkempt coat, highly sluggish response to external stimuli, severe weakened ability to withstand external stressors
LPS+FXR inhibitor 2	Severe depression, minimal activity, severely diminished appetite and fluid intake, watery stools, significantly reduced urine output, dull and unkempt coat, highly sluggish response to external stimuli, severe weakened ability to withstand external stressors
LPS+FXR inhibitor 3	Severe depression, minimal activity, severely diminished appetite and fluid intake, watery stools, significantly reduced urine output, dull and unkempt coat, highly sluggish response to external stimuli, severe weakened ability to withstand external stressors
LPS+FXR inhibitor 4	Severe depression, minimal activity, severely diminished appetite and fluid intake, watery stools, significantly reduced urine output, dull and unkempt coat, highly sluggish response to external stimuli, severe weakened ability to withstand external stressors
LPS+FXR inhibitor 5	Severe depression, minimal activity, severely diminished appetite and fluid intake, watery stools, significantly reduced urine output, dull and unkempt coat, highly sluggish response to external stimuli, severe weakened ability to withstand external stressors
LPS+FXR inhibitor 6	Severe depression, minimal activity, severely diminished appetite and fluid intake, watery stools, significantly reduced urine output, dull and unkempt coat, highly sluggish response to external stimuli, severe weakened ability to withstand external stressors

### 3.2 FXR Activation Triggered FGF15/FGFR4 Pathway Activation

**Figure 2:**
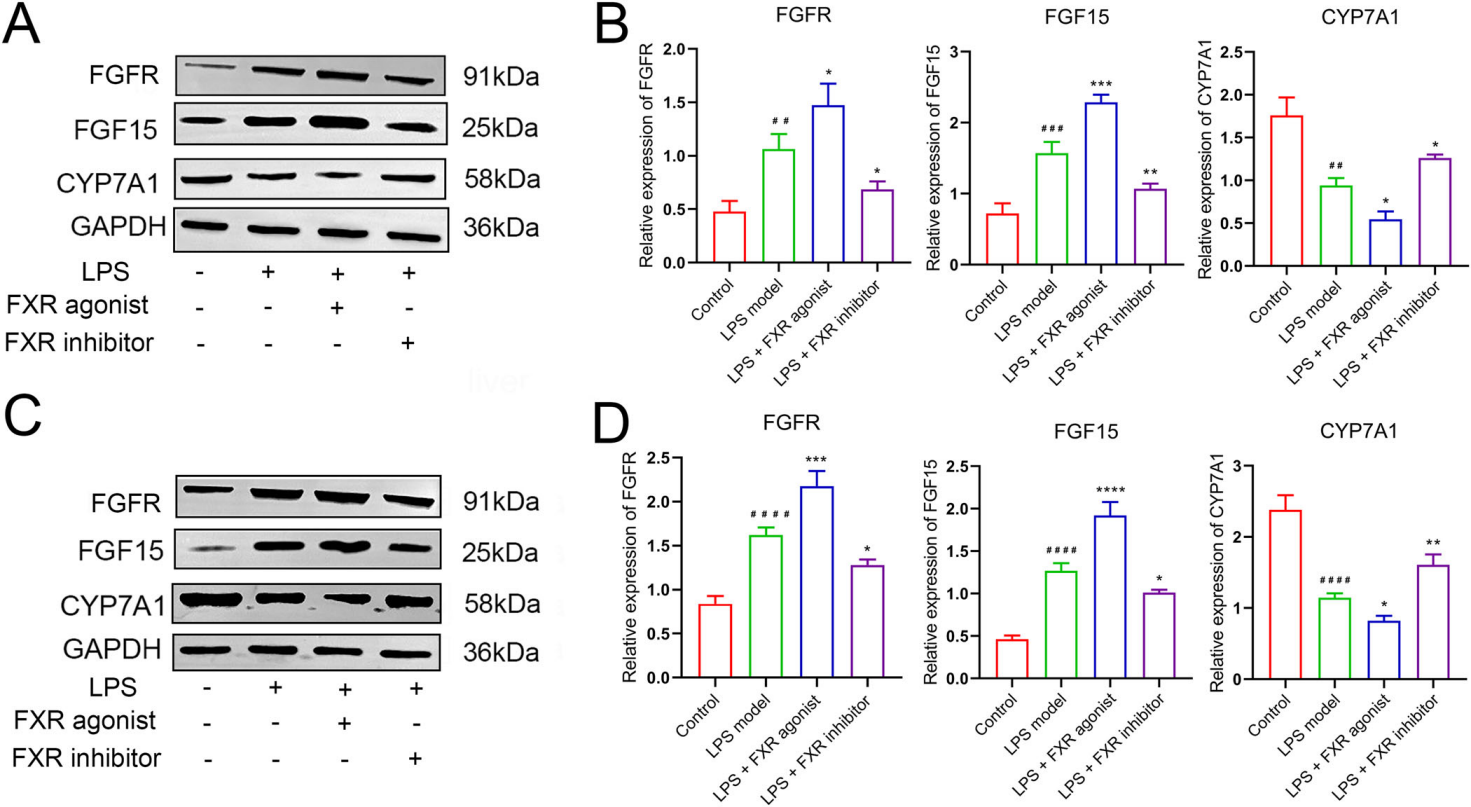
Expression of relative proteins in the FGF15/FGFR4 pathway after FXR activation detected by Western blot. (A–B) FGF15, FGFR, and CYP7A1 protein expressions in the ileum tissue. (C–D) FGF15, FGFR, and CYP7A1 protein expressions in the liver tissue. ^##^ *p* < 0.01, ^###^ *p* < 0.001, ^####^ *p* < 0.001, control versus LPS model; *p < 0.05, **p < 0.01, ***p < 0.001, ****p < 0.0001, LPS model versus LPS + FXR agonist/inhibitor. FXR, farnesoid X receptor; H&E, hematoxylin and eosin; LPS, lipopolysaccharide.

Subsequently, the mRNA expression levels of the key genes in the FGF15/FGFR4 pathway were investigated with qRT‐PCR. CYP7A1 expression level in the ileum tissue in the agonist treatment group was significantly lower (*p* < 0.05), whereas those in the inhibitor treatment and control groups were significantly higher compared with the LPS model group (*p *< 0.05). Conversely, FGF15 and FGFR expression trends in the four groups were inversely correlated with those of CYP7A1 (Figure 1E). Parallel expression trends of three mRNAs were revealed likewise in the liver tissue (Figure 1F). Similarly, Western Blot analysis revealed a high degree of concordance between the protein expression patterns of FGF15, FGFR, and CYP7A1 in the ileum and liver tissues with their corresponding mRNA expressions (Figure 2). Thus, the FXR agonist can induce FGF15/FGFR4 pathway activation, thereby ameliorating sepsis‐induced hepatic injury according to our results.

### 3.3 FXR Activation Promoted Bile Acid Metabolism and Attenuated Inflammatory Responses

**Figure 3:**
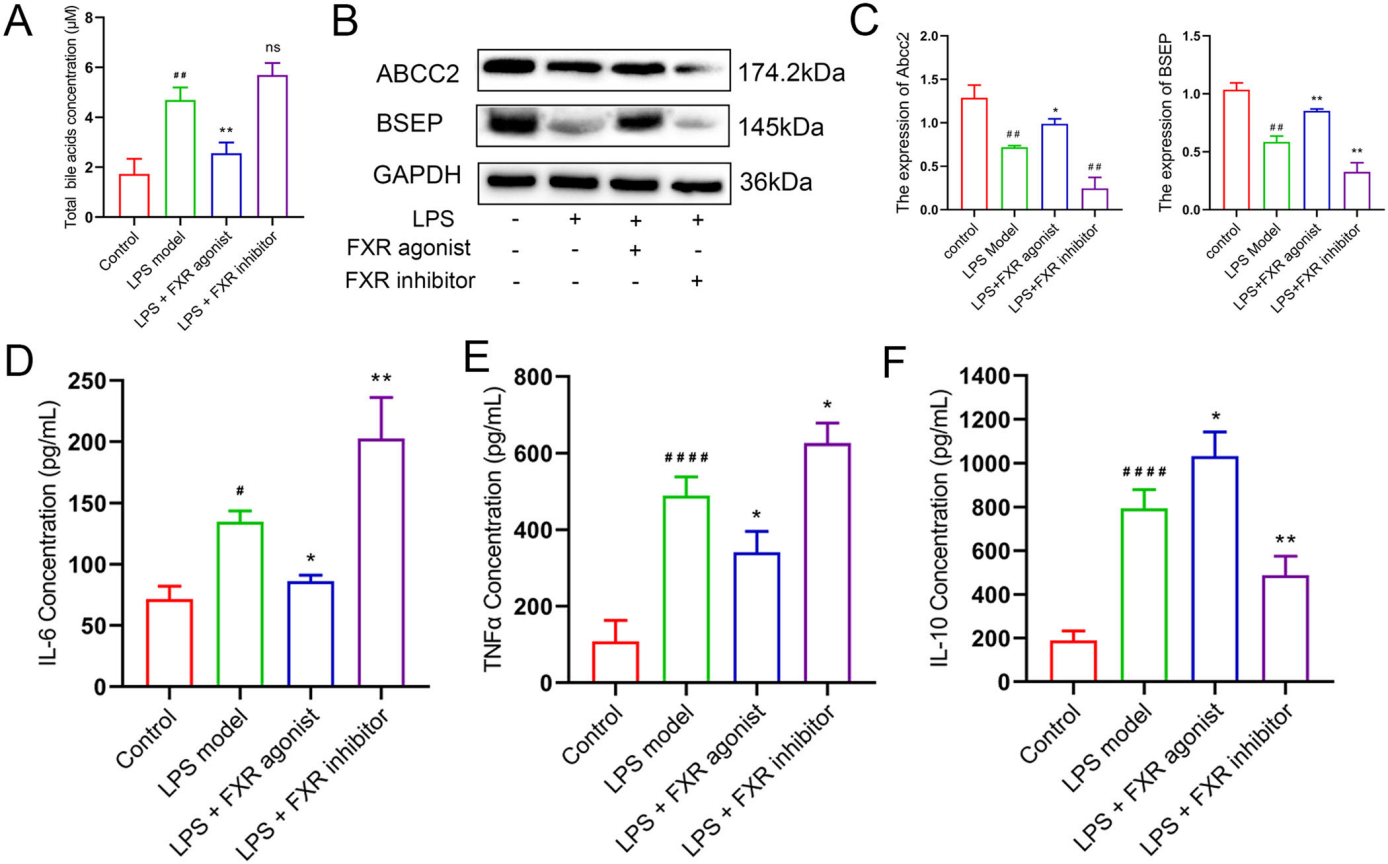
Variations of hematological biomarkers after FXR activation. (A) Total bile acid concentration of the control, LPS model, LPS + agonist, and LPS + inhibitor groups. (B–C) The expression of ABCC2 and BSEP was examined using western blot. (D) IL‐6 concentration of the control, LPS model, LPS + agonist, and LPS + inhibitor groups detected with ELISA. (E) TNF‐α concentration of the control, LPS model, LPS + agonist, and LPS + inhibitor groups detected with ELISA. (F) IL‐10 concentration of the control, LPS model, LPS + agonist, and LPS + inhibitor groups detected with ELISA. ^#^ *p* < 0.05, ^###^ *p* < 0.01, ^####^ *p* < 0.001, control versus LPS model; *p < 0.05, **p < 0.01, LPS model versus LPS + FXR agonist/inhibitor. ELISA, enzyme‐linked immunosorbent assay; FXR, farnesoid X receptor; LPS, lipopolysaccharide; ns, no statistical significantly.

The contents of total bile acid and cytokines were identified to investigate the effects of FXR activation through the blood extracted from mice. The results of total bile acid concentration determination indicated that the FXR agonist could significantly decrease the bile acid content (*p *< 0.01), which demonstrated the capacity of FXR agonist in improving the metabolism of bile acids and potential preventive capacity of cholestasis (Figure 3A). Meanwhile, examination of the BA production indicated ABCC2 and BSEP were decreased in liver induced by LPS, which were reversed by FXR agonist (*p *< 0.01, Figure 3B,C). The decreasion of ABCC2 and BSEP led to inflammatory response induced by LPS. Thus, IL‐6, IL‐10, and TNF‐α concentrations in the serum of mice were investigated via the ELISA experiment procedure. Figure 3D illustrates that IL‐6 was significantly decreased after FXR activation (*p* < 0.05) and increased after FXR inhibition (*p *< 0.01). The concentration variation of TNF‐α indicated a similar trend, with all the differences being significant compared with the LPS model group (Figure 3E). Interestingly, disparate variation trends were revealed in IL‐10, indicating that FXR agonist treatment contributed to the content elevation of IL‐10 (Figure 3F). The mechanism may be attributed to the FXR agonist treatment that inhibits the excessive inflammatory response and protects cells via improving IL‐10 activation through comprehensive analysis.

### 3.4 Targeted Quantitative Determination of Bile Acid Contents

A robust quantitative analysis method was developed with LC‐MS to precisely determine 41 bile acid species in serum samples, and a total of 12 serum samples were identified. Table 2 exhibits that all the correlation coefficients of linear regression equations for bile acids were > 0.99 and the retention time, linear range, and limit of quantification of each bile acid were investigated. The recovery rates of all 41 bile acids were then calculated according to the peak area of standard samples. The results indicated that the recovery rates were 82%–120.8%, indicating the robustness, reliability, and suitability of this method for sample detection (Table 3). Eventually, the concentrations of the target in the serum samples were identified according to the standard curve and the signal intensity obtained during quantitative detection (Table 4).

**Table 2:** Linear regression equations and their quantitative limits.

Bile acid type	Retention time (min)	Linear regression equation	Correlation coefficient (*r*)	linear range (ng/mL)	Limit of quantification (ng/mL)
DHLCA	30.80	y = 15.76984 x + −0.01945	0.99203	1–8000	1
isoLCA	28.55	y = 7.86051 x + −0.37420	0.99120	5–8000	5
LCA	30.05	y = 11.90091 x + −0.53871	0.99520	5–8000	5
AILCA	28.23	y = 3.76249 x + −0.04032	0.99903	5–2000	5
23norDCA	19.72	y = 2.20310 x + −0.01513	0.99762	1–8000	1
apoCA	21.81	y = 5.10358 x + 0.03350	0.99494	5–8000	5
7‐ketoLCA	18.96	y = 6.36355 x + 0.22366	0.99525	1–8000	1
12‐ketoLCA	20.31	y = 5868.63922 x + 14654.87315	0.99743	1–8000	1
isoDCA	28.20	y = 4.45908 x + −0.03330	0.99627	1–8000	1
DCA	24.01	y = 2.24410 x + −0.00581	0.99123	1–8000	1
HDCA	15.87	y = 8.26798 x + 0.09406	0.99902	1–8000	1
UDCA	15.43	y = 4467.39990 x + 5631.82327	0.99219	1–8000	1
CDCA	23.23	y = 3578.08850 x + 3463.58739	0.99853	5–8000	5
DHCA	8.59	y = 0.07089 x + −0.17300	0.99560	5–8000	5
3‐H‐7,12‐DKCA	7.71	y = 2223.79955 x + −1829.15465	0.99490	5–8000	5
6,7‐diketoLCA	19.41	y = 1010.87456 x + 6794.10613	0.99926	10–8000	10
3‐DHCA	13.81	y = 13.48855 x + 0.00351	0.99612	0.5–8000	0.5
7‐KHCA	10.69	y = 23.75660 x + 0.11720	0.99515	5–8000	5
12‐OCDCA	10.94	y = 3791.57624 x + 2641.37724	0.99101	0.5–8000	0.5
ACA	13.43	y = 3.25811 x + −0.01527	0.99499	1–8000	1
UCA	7.00	y = 346.55505 x + −169.74349	0.99637	1–8000	1
CA	14.52	y = 1.02222 x + −0.00196	0.99792	1–8000	1
B‐MCA	11.04	y = 128.97257 x + −513.04538	0.99684	5–2000	5
HCA	14.51	y = 17.07717 x + 0.04779	0.99936	1–8000	1
a‐MCA	11.05	y = 0.08981 x + 0.12664	0.99714	1–8000	1
GLCA	25.28	y = 4.69981 x + −0.03755	0.99381	1–8000	1
GDCA	17.46	y = 1776.39466 x + −1275.33641	0.99871	1–8000	1
GCDCA	16.01	y = 1538.97838 x + −920.15748	0.99829	1–8000	1
GUDCA	9.86	y = 0.00459 x + −0.00470	0.99564	5–8000	5
GHDCA	10.13	y = 2044.04387 x + −1062.15712	0.99361	1–2000	1
GDHCA	3.87	y = 0.00255 x + −0.00931	0.99900	5–8000	5
GCA	10.10	y = 0.00731 x + −9.01591e‐4	0.99808	10–8000	10
GHCA	7.77	y = 0.02344 x + −0.02067	0.99847	1–8000	1
TLCA	27.97	y = 1.61854 x + −0.12961	0.99488	10–8000	10
TDCA	21.00	y = 1.58038 x + −0.09165	0.99776	5–8000	5
THDCA	10.79	y = 0.01282 x + −0.14473	0.99898	10–8000	10
TCDCA	18.38	y = 0.00297 x + −0.01093	0.99752	5–8000	5
TUDCA	10.67	y = 770.19809 x + −3711.83820	0.99961	5–8000	5
T‐B‐MCA	5.89	y = 449.32928 x + −3711.50625	0.99994	10–8000	10
T‐a‐MCA	7.63	y = 98.40021 x + −310.09353	0.99454	5–8000	5
TCA	11.24	y = 344.57970 x + −1789.85630	0.99846	5–8000	5

**Table 3:** Recovery rates of 41 types of bile acids.

Bile acid types	QC‐1 samples concentration (ng/mL)	QC‐2 samples concentration (ng/mL)	Actual added concentration (ng/mL)	Average recovery (%)
DHLCA	2.47E + 02	2.05E + 02	250	90.3
isoLCA	2.44E + 02	2.06E + 02	250	90.1
LCA	2.29E + 02	1.81E + 02	250	82
AILCA	2.60E + 02	2.31E + 02	250	98.18
23norDCA	2.43E + 02	2.48E + 02	250	98.18
apoCA	2.48E + 02	2.71E + 02	250	103.7
7‐ketoLCA	2.53E + 02	2.52E + 02	250	100.96
12‐ketoLCA	3.07E + 02	2.97E + 02	250	120.8
isoDCA	2.66E + 02	2.42E + 02	250	101.7
DCA	2.62E + 02	2.62E + 02	250	104.92
HDCA	2.43E + 02	2.65E + 02	250	101.6
UDCA	2.44E + 02	2.50E + 02	250	98.86
CDCA	2.87E + 02	2.86E + 02	250	114.54
DHCA	2.79E + 02	2.65E + 02	250	108.76
3‐H‐7,12‐Dkca	2.71E + 02	2.74E + 02	250	109.08
6,7‐diketoLCA	2.78E + 02	3.08E + 02	250	117.22
3‐DHCA	2.69E + 02	2.85E + 02	250	110.8
7‐KHCA	2.78E + 02	2.72E + 02	250	110.04
12‐OCDCA	2.78E + 02	3.06E + 02	250	116.84
ACA	2.47E + 02	2.73E + 02	250	103.88
UCA	2.68E + 02	2.78E + 02	250	109.24
CA	2.59E + 02	2.63E + 02	250	104.4
B‐MCA	2.71E + 02	2.67E + 02	250	107.58
HCA	2.57E + 02	2.70E + 02	250	105.38
a‐MCA	2.77E + 02	2.79E + 02	250	111.3
GLCA	2.55E + 02	2.57E + 02	250	102.38
GDCA	2.59E + 02	2.48E + 02	250	101.46
GCDCA	2.78E + 02	2.67E + 02	250	108.92
GUDCA	2.21E + 02	2.55E + 02	250	95.14
GHDCA	2.53E + 02	2.60E + 02	250	102.5
GDHCA	2.77E + 02	2.80E + 02	250	111.42
GCA	2.62E + 02	2.55E + 02	250	103.2
GHCA	2.49E + 02	2.52E + 02	250	100.14
TLCA	2.49E + 02	2.35E + 02	250	96.92
TDCA	2.39E + 02	3.06E + 02	250	108.94
THDCA	2.37E + 02	2.75E + 02	250	102.36
TCDCA	2.43E + 02	2.58E + 02	250	100.16
TUDCA	2.76E + 02	2.95E + 02	250	114.14
T‐B‐MCA	2.63E + 02	2.51E + 02	250	102.62
T‐a‐MCA	2.36E + 02	3.06E + 02	250	108.38
TCA	2.69E + 02	2.74E + 02	250	108.52

**Table 4:** Concentrations and analysis of 41 types of bile acids in the serum samples.

Bile acid type	Sample group								
	QC1	NC	LPS	*p* value (LPS vs. NC)	QC2	FXR agonist	*p* value (FXR agonist vs. LPS)	FXR inhibitor	*p* value (FXR inhibitor vs. LPS)
DHLCA	246.5	0.05881	0.03724	0.031021683	205	0.0344133	0.727753856	0.0827	0.072022861
isoLCA	244.1	0.0956067	0.08089	0.387264107	206.4	0.1038067	0.231574946	0.0891433	0.536931201
LCA	228.6	0.1518333	0.0961767	0.147177763	181.4	0.1559333	0.177000703	0.1256667	0.037505477
AILCA	260.4	0.0473267	0.11702	0.297837756	230.5	0.0873267	0.646437528	0.1365333	0.856808455
23norDCA	243.2	0.4498333	0.1701	0.257598716	247.7	0.1946	0.65319671	0.1718667	0.954571358
apoCA	248	0.1364233	0.1231533	0.85323216	270.5	0.2339	0.368687764	0.7396333	0.145626194
7‐ketoLCA	253.1	0.0713367	0.0575167	0.781188678	251.7	0.2087	0.267273688	0.0411	0.732010372
12‐ketoLCA	306.8	6.7403333	11.826667	0.139798756	297.2	24.823333	0.241275097	33.123333	0.231946459
isoDCA	266.4	0.00984	0.0085853	0.401726916	242.1	0.0078083	0.452638211	0.0082633	0.770713287
DCA	262.2	3.6216667	4.3593333	0.663267118	262.4	5.9776667	0.662270021	4.3466667	0.995180375
HDCA	243	4.488	2.3096667	0.362103416	265	5.7213333	0.47443714	8.0797333	0.319977509
UDCA	244	517.33333	207.06667	0.369271071	250.3	152.05333	0.356814626	242.10333	0.793925357
CDCA	286.6	234.4	80.48	0.089871542	286.1	147.92	0.458575285	247.71	0.203532917
DHCA	278.5	4.2343333	33.206667	0.044870425	265.3	27.523333	0.663457804	54.073333	0.075212512
3‐H‐7,12‐Dkca	271.2	3.3753333	6.2146667	0.181282731	274.2	7.1633333	0.674174206	12.246667	0.078273574
6,7‐diketoLCA	278.3	112.49	N/A	N/A	307.8	0.74645	N/A	88.97	0.171321079
3‐DHCA	269.3	0.0101922	0.0040267	0.420946175	284.7	0.0096833	0.170307402	0.0360817	0.188308081
7‐KHCA	278.1	0.8297667	0.3209333	0.468700473	272.1	0.8762667	0.243412749	1.3064667	0.353738603
12‐OCDCA	278.4	82.648333	32.403333	0.450665686	305.8	54.884333	0.42902108	53.036667	0.50833941
ACA	246.7	0.0633167	0.0426367	0.466559839	272.7	0.0522067	0.678677721	0.1283933	0.289034272
UCA	268.2	24.262333	9.6033333	0.459447481	278	9.6883333	0.987976401	16.43	0.330111923
CA	258.9	8.2906667	5.1323333	0.545490089	263.1	7.6326667	0.429584531	12.373	0.260869375
B‐MCA	270.7	1933.9	1010.7	0.536996901	267.2	684.66667	0.479286291	1090.3	0.909802167
HCA	257.3	8.552	5.214	0.538138432	269.6	7.6676667	0.44742695	12.489	0.258886143
a‐MCA	277.4	1135.1667	386.43333	0.38073954	279.1	287.16333	0.587267726	496.26667	0.733325265
GLCA	254.5	0.0081143	0.0080923	0.656111352	257.4	0.0080763	0.756874367	0.0080773	0.789416537
GDCA	259.4	0.7772	1.1301333	0.234464933	247.9	3.5469	0.204237544	1.2476333	0.706377826
GCDCA	278	0.6922333	0.7312	0.537992558	266.6	0.9366667	0.184902459	0.7839333	0.581311454
GUDCA	221.2	1.2453333	1.6496667	0.493365348	254.5	2.5383333	0.345775703	1.5343333	0.838986701
GHDCA	252.7	0.6910667	0.7857667	0.579791315	259.8	0.7761	0.960795031	0.8416333	0.766329693
GDHCA	277.3	0	0	N/A	279.8	2.6156667	0.117073119	1.2233333	0.373900966
GCA	261.5	5.544	6.3726667	0.699210413	254.5	10.613333	0.180335415	4.1066667	0.24947513
GHCA	248.5	0.9131333	0.2993333	0.109841402	252.2	0.9363	0.101424107	N/A	N/A
TLCA	249.3	0.1251333	0.0947633	0.020606566	235.3	0.099	0.542263748	0.1038233	0.265541014
TDCA	238.5	0.4356	4.0123333	0.007635433	306.2	16.769	0.069914767	9.0786667	0.005463827
THDCA	236.5	31.49	42.853333	0.242816045	275.3	357.26667	0.075832606	178.9	0.018436809
TCDCA	243.1	101.36333	644.3	0.241009831	257.7	455.76667	0.696420152	567.1	0.859760642
TUDCA	275.9	43.433333	103.81667	0.007291078	294.8	925.76667	0.071246989	59.037	0.461202671
T‐B‐MCA	262.6	384.2	704.83333	0.108007201	250.5	2459.6667	0.028081275	578.66667	0.611889843
T‐a‐MCA	236.1	3890.3333	19180	0.02624272	305.8	4685	0.013291247	717.36667	0.018536963
TCA	268.5	985.73333	1134.2	0.802386209	274.1	1008.6667	0.838864736	684.43333	0.391220794

*Note:* Sample name representation: QC, quality control; NC, control; LPS, LPS model.

### 3.5 Metabolism Alterations in Each Bile Acid Type

**Figure 4:**
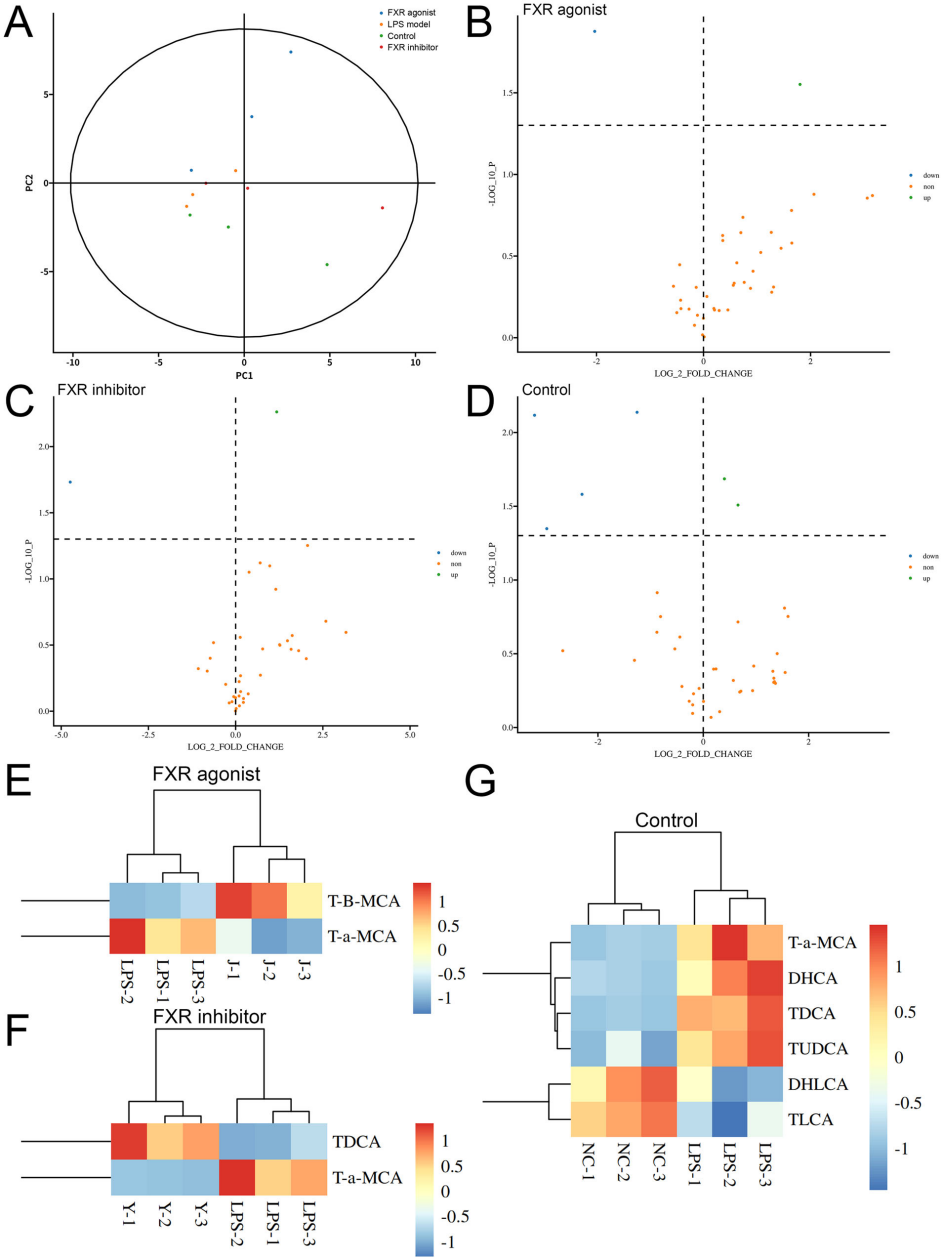
Metabolomics analysis results of bile acid concentration variations. (A) PCA plot of the samples in the control, LPS model, LPS + agonist, and LPS + inhibitor groups. (B) Volcano plot of bile acids with various concentrations following the FXR agonist treatment. (C) Volcano plot of bile acids with various concentrations following the FXR inhibitor treatment. (D) Volcano plot of bile acids with various concentrations between the control and the LPS model groups. (E) Heat map of bile acids with various concentrations following the FXR agonist treatment. (F) Heat map of bile acids with different concentrations following the FXR inhibitor treatment. (G) Heat map of bile acids with various concentrations between the control and the LPS model groups. FXR, farnesoid X receptor; LPS, lipopolysaccharide; PCA, principal component analysis.

Bioinformatics analysis was conducted to perform further crucial investigations after obtaining the quantitative data. The PCA indicated that the control, LPS model, FXR agonist, and FXR inhibitor groups separated clearly, with all samples in the 95% confidence interval (Figure 4A). The subsequent differential analysis revealed that several bile acid types demonstrated significantly different concentrations, with the red dots representing bile acids that have been upregulated (fold change of > 1 and *p *< 0.05) and the blue dots representing bile acids (fold change of < 1 and *p *< 0.05) (Figure 4B–D). Subsequently, all the differential bile acids were further investigated for significant differences in bile acid expression. T‐B‐MCA was upregulated in the FXR agonist group, whereas TDCA was upregulated in the inhibitor group (Figure 4E–G). Interestingly, T‐a‐MCA, as a competitive natural FXR antagonist, was downregulated after both agonist and inhibitor treatment, which may be attributed to the competitive effect. Supporting Information S1: Figure 1 exhibits the box plots of each bile acid type concentration analysis, with *p *< 0.05 representing statistical significance.

## 4 Discussion

The mortality of sepsis remains unacceptably high despite its sophisticated and precise diagnostic criteria and progressively improving treatment efficacy. Sepsis encompasses a spectrum of complications, in which cholestasis demonstrates an earlier symptom compared with other organ failure [22]. Furthermore, cholestasis may become a potential diagnostic and prognostic biomarker in sepsis due to its relative ease in disease detection [23]. Thus, the adequate attention given to sepsis‐induced cholestasis, along with timely diagnosis and treatment, may effectively prevent further deterioration of sepsis and its associated complications.

The current study conducted a comprehensive exploration to confirm the mechanism of abnormal bile acid metabolism induced by sepsis. The normal bile acid metabolism is undoubtedly related to the involvement of the physiological enterohepatic circulation. However, the liver, as widely acknowledged in sepsis conditions, is a central immune organ in regulating host defenses and is extremely vulnerable to bacterial endotoxin‐induced injury and ischemic damage caused by sepsis [24]. Besides, the significance of the liver in various metabolism procedures is undoubted, and hepatic damage inevitably causes aberrations in bile acid metabolism. The sepsis mouse model was developed based on these characteristics of disease symptoms for subsequent exploration via receiving the intraperitoneal injection of LPS, with the liver tissue being injured and higher bile acid concentration in serum.

Numerous studies have revealed sepsis‐associated bile metabolism dysregulation as a highly intricate abnormal metabolic process, involving multiple mechanisms, pathways, and aberrant tissues. The prevailing viewpoint for this disorder at present is about NLRP3 inflammasome [25], whereas, few reports pay attention to the classic FXR/FGF15/FGFR bile acid metabolism pathway. Additionally, the report of cholestasis induced by NAFLD [26, 27] indicated that bile acid production was significantly increased during the NAFLD condition and the proportion of FXR antagonistic bile acid was elevated, thereby explaining hepatic FXR and FGFR4 signaling suppression. Therefore, our study postulated that this pathway would similarly demonstrate aberrations in the context of sepsis. The activation and inhibition treatments were conducted for FXR in the sepsis model, with the variation in hepatic injury, total bile acid concentrations, and the expression level of key genes and proteins detected and analyzed, to validate our hypothesis. The aforementioned investigation revealed the involvement of the FXR pathway in aberrant bile acid metabolic processes. Moreover, the activation treatment of FXR has been demonstrated to ameliorate liver injury and cholestasis, which is consistent with previous reports on abnormal metabolism in other disease conditions, encompassing colorectal cancer [28, 29, 30] and cholesterol gallstones [31].

FXR in the FXR/FGF15/FGFR4 pathway is the upstream regulatory receptor of the metabolism regulations of bile acids in the enterohepatic circulation [32, 33]. Its highest expression locations are concentrated in the liver and intestine, with bile acids confirmed to be the natural ligands [34]. Excessive bile acid will activate the FXR to regulate the downstream reabsorption under normal regulatory circumstances [35, 36]. However, bile acid substantially accumulates after sepsis, which causes the failure in efficient reabsorption of the surplus bile acid, despite the presence of limited activated FXR. The emergence and utilization of synthetic FXR agonists may potentially contribute to alleviating this terrible condition; thus, our attention was subsequently oriented to the effect of FXR agonist treatment on downstream bile acid metabolism regulation. Mouse‐derived FGF15 (homologous to human FGF19) acts as the endogenous hormone that circulates through the enterohepatic circulation. Several studies have revealed that activated FXR promotes FGF15/19 protein synthesis and secretion by regulating FGF15/19 gene transcription through a series of processes [37]. Subsequently, FGF15/19 is delivered to the liver through the portal vein and combined with the receptor complex of FGFR4‐β‐klotho (KLβ), thereby activating the FGFR4 [38, 39, 40, 41]. FGFR4 is a significant FGFR family isoform, which is highly detected in the liver [42] and demonstrates the FGF binding activity in the metabolism regulatory procedure. The negative feedback regulatory mechanism of CYP7A1 gene expression is triggered after the binding of FGF and FGFR, thereby reducing the production of the rate‐limiting enzyme CYP7A1 in the classical pathway of bile acid synthesis [19, 43, 44]. After the regulatory process, we conducted a comprehensive investigation into the expression levels of genes and coding proteins of the aforementioned key factors within the pathway after FXR agonist and inhibitor treatment, respectively. The expression levels of all genes and proteins we collected in our experimental determination were significantly different after FXR agonist treatment and consistent with results from previous studies. Furthermore, the result of the total bile acid concentration determination was significantly decreased after FXR agonist treatment compared with the sepsis model. The above findings conclude that activating FXR and its downstream pathways by FXR agonists effectively improves bile acid metabolism, thereby restoring bile acid levels to normal and demonstrating a certain alleviating effect on liver injury, which may demonstrate the potential significance of FXR agonists in sepsis intervention.

Inflammatory response dysregulation represents another pivotal manifestation of sepsis. Previous evidence indicated that the exaggerated inflammatory response manifested early period of sepsis progression [45], consistent with the onset of cholestasis. Therefore, the inflammatory factors concentrations were investigated after FXR agonist treatment to examine whether FXR agonists can attenuate inflammatory response. Comfortingly, significant alterations in the serum IL‐6, IL‐10, and TNF‐α concentrations were observed in the mouse model after FXR activation, which may indicate the inflammation inhibition effect of the FXR agonist.

Omics detection technology has appeared as a powerful tool for elucidating the pathogenic mechanisms underlying diseases and facilitating precise disease diagnosis and treatment with the advancement of medical research toward high‐throughput and high‐precision methodologies. As far back as a decade ago, Seymour and colleagues conducted a study utilizing metabolomics techniques to investigate the disparities in bile acid metabolites between patients with sepsis and healthy volunteers within the GenIMS cohort study project [46]. Subsequently, numerous studies have concentrated on the global metabolic profile of metabolites, such as bile acids, in sepsis patients and endeavored to establish prognosis models for sepsis patients based on the detected bile acid levels [47, 48]. The current study conducted the metabolomics analysis to reveal the precise quantitative alterations in each bile acid and obtain a comprehensive spectrum of each type of bile acid content alteration in septic mice, as well as in septic mice following intervention with FXR agonists or inhibitors. In comparison to the normal control group, six distinct types of bile acids exhibiting significant differences in concentration were identified in mice with sepsis induced by LPS. Furthermore, two kinds of bile acids demonstrated differential expression between the agonist/inhibitor treatment and sepsis mouse model, respectively. Interestingly, Tauro α‐Muricholic acid (T‐α‐MCA) expression was downregulated after either an FXR activator or an FXR inhibitor treatment. T‐α‐MCA is a competitive and reversible endogenous inhibitor of FXR [49]; thus, we preliminarily infer that its expression or activity may be diminished due to competitive effects or transformed into other compounds through other mechanisms under a more powerful synthetic activator/inhibitor intervention. However, elucidating the specific mechanism underlying this phenomenon poses an interesting and challenging research, which requires further investigation in subsequent works.

## 5 Conclusions

Our research identified FXR as a promising target for treating sepsis‐induced cholestasis, and the agonist of FXR demonstrates the potential preventive applications in treating sepsis, which may facilitate more precise intervention of sepsis in the future, thereby mitigating hospitalization duration, mortality, and associated societal burden of sepsis.

## Author Contributions

**Ziyang Zhou:** conceptualization, writing–original draft. **Dan Xu:** data curation, methodology. **Liou Huang:** data curation, methodology. **Yuhui Cui:** formal analysis, investigation. **Hui Chen:** formal analysis, investigation. **Jianguo Tang:** writing–review and editing.

## Ethics Statement

Animal experiments have been approved by the ethics committee(m20240810).

## Consent

The authors have nothing to report.

## Conflicts of Interest

The authors declare no conflicts of interest.

## Supporting information

Supporting information.

Supporting information.

Supporting information.

Supporting information.

Supporting information.

## Data Availability

The data sets used and/or analyzed during the current study are available from the corresponding author upon reasonable request.
