# Supplementary figures and images for: Farnesoid X Receptor Regulated Sepsis‐Induced Abnormal Bile Acid Metabolism via the Fibroblast Growth Factor 15/Fibroblast Growth Factor Receptor 4 Pathway
Source: Immun Inflamm Dis. 2025 Apr 7;13(4):e70155. doi: 10.1002/iid3.70155 (PMC11973727; doi:10.1002/iid3.70155)

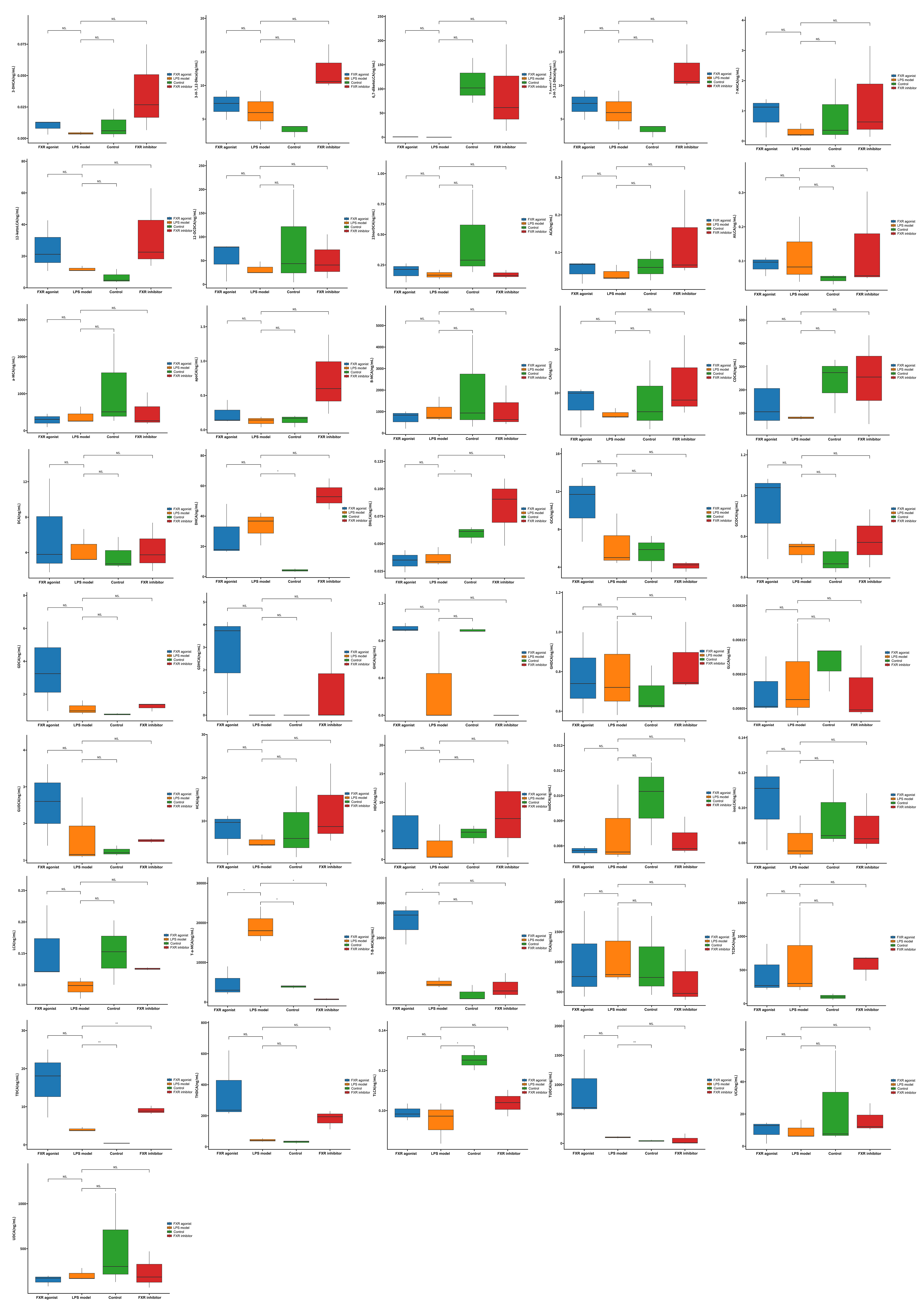

Supplement: Supplementary file 1 — Supporting information. [file IID3-13-e70155-s005.jpg]
