# Supplementary material for: Farnesoid X Receptor Regulated Sepsis‐Induced Abnormal Bile Acid Metabolism via the Fibroblast Growth Factor 15/Fibroblast Growth Factor Receptor 4 Pathway
Source: Immun Inflamm Dis. 2025 Apr 7;13(4):e70155. doi: 10.1002/iid3.70155 (PMC11973727; doi:10.1002/iid3.70155)
Supplement: Supplementary file 2 — Supporting information. [file IID3-13-e70155-s001.docx]

**Supplemental Table 1** Primers sequences for qRT-PCR.

| Gene | | Forward primer | Reverse primer |
| --- | --- | --- | --- |
| FGF15 | ATGGCGAGAAAGTGGAACGG | CTGACACAGACTGGGATTGCT |  |
| FGFR | TAATACCACCGACAAGGAAATGG | TGATGGGAGAGTCCGATAGAGT |  |
| CYP7A1 | GGGATTGCTGTGGTAGTGAGC | GGTATGGAATCAACCCGTTGTC |  |
| GAPDH | GACAGCCGCATCTTCTTGTG | AATCCGTTCACACCGACCTT |  |
|  | | |  |
