# Supplementary material for: Farnesoid X Receptor Regulated Sepsis‐Induced Abnormal Bile Acid Metabolism via the Fibroblast Growth Factor 15/Fibroblast Growth Factor Receptor 4 Pathway
Source: Immun Inflamm Dis. 2025 Apr 7;13(4):e70155. doi: 10.1002/iid3.70155 (PMC11973727; doi:10.1002/iid3.70155)
Supplement: Supplementary file 3 — Supporting information. [file IID3-13-e70155-s003.docx]

**Supplemental Table 2** Types of bile acid standards.

| Abbreviation | Full name |
| --- | --- |
| 23norDCA | 23-Nordeoxycholic acid |
| apoCA | Apocholic acid |
| TDCA | Taurodeoxycholic acid |
| TLCA | Taurolithocholic acid |
| Β-MCA | β-Muricholic acid |
| GDHCA | Glycodehydrocholic acid |
| 3-DHCA | 3-Dehydrocholic acid |
| THDCA | Taurohyodeoxycholic acid |
| 7-KHCA | 7-Ketodeoxycholic acid |
| isoDCA | Isodeoxycholic acid |
| GLCA | Glycolithocholic acid |
| DHLCA | Dehydrolithocholic acid |
| isoLCA | Isolithocholic acid |
| T-Β-MCA | Tauro β-Muricholic acid |
| T-α-MCA | Tauro α-Muricholic acid |
| 12-OCDCA | 12-Oxochenodeoxycholic Acid |
| TCDCA | Taurochenodeoxycholic acid |
| 7-ketoLCA | 7-Ketolithocholic acid |
| TCA | Taurocholic acid |
| DHCA | Dehydrocholic acid |
| LCA | Lithocholic acid |
| HDCA | Hyodeoxycholic acid |
| CA | Cholic acid |
| GCA | Glycocholic acid |
| GDCA | Glycodeoxycholic acid |
| TUDCA | Tauroursodeoxycholic acid |
| GUDCA | Glycoursodeoxycholic acid |
| DCA | Deoxycholic acid |

Continued table

| GCDCA | Glycochenodeoxycholic acid |
| --- | --- |
| ACA | Allocholic acid |
| GHDCA | Glycohyodeoxycholic acid |
| CDCA | Chenodeoxycholic acid |
| UDCA | Ursodeoxycholic acid |
| AILCA | Alloisolithocholic acid |
| HCA | Hyocholic acid |
| GHCA | Glycohyocholic acid |
| 12-ketoLCA | 12-Ketolithocholic acid |
| UCA | Ursocholic acid |
| 3-H-7,12-DKCA | 3-Hydroxy-7,12-diketocholanoic acid |
| α-MCA | α-Muricholic acid |
| 6,7-diketoLCA | 6,7-diketolithocholic acid |
