# Supplementary material for: Farnesoid X Receptor Regulated Sepsis‐Induced Abnormal Bile Acid Metabolism via the Fibroblast Growth Factor 15/Fibroblast Growth Factor Receptor 4 Pathway
Source: Immun Inflamm Dis. 2025 Apr 7;13(4):e70155. doi: 10.1002/iid3.70155 (PMC11973727; doi:10.1002/iid3.70155)
Supplement: Supplementary file 4 — Supporting information. [file IID3-13-e70155-s004.docx]

**Supplemental Table 3** Gradient of mobile phase in liquid chromatography.

| Time (min) | A phase (%) | B phase (%) |
| --- | --- | --- |
| 0 | 75 | 25 |
| 4 | 75 | 25 |
| 9 | 70 | 30 |
| 12 | 67 | 33 |
| 18 | 65 | 35 |
| 26 | 50 | 50 |
| 33 | 25 | 75 |
| 35 | 5 | 95 |
| 35.5 | 5 | 95 |
| 36 | 75 | 25 |
| 38 | 75 | 25 |
