# Supplementary material for: Farnesoid X Receptor Regulated Sepsis‐Induced Abnormal Bile Acid Metabolism via the Fibroblast Growth Factor 15/Fibroblast Growth Factor Receptor 4 Pathway
Source: Immun Inflamm Dis. 2025 Apr 7;13(4):e70155. doi: 10.1002/iid3.70155 (PMC11973727; doi:10.1002/iid3.70155)
Supplement: Supplementary file 5 — Supporting information. [file IID3-13-e70155-s002.docx]

**Supplemental Table 4** Mass spectrometry acquisition parameters.

| Determinand | Parent ion | Daughter ion | DP (V) | CE (V) |
| --- | --- | --- | --- | --- |
| 23norDCA | 377.3 | 331.3 | -75.8 | -40 |
| apoCA | 389.2 | 389.2 | -55.3 | -10 |
| TDCA | 498.3 | 124 | -89.5 | -59 |
| TLCA | 482.3 | 124 | -80.4 | -58 |
| B-MCA | 407.3 | 371 | -105.1 | -40 |
| GDHCA | 458.2 | 388.2 | -80 | -35 |
| 3-DHCA | 405.2 | 289.2 | -80.4 | -45 |
| THDCA | 498.3 | 124.1 | -94.1 | -57 |
| 7-KHCA | 405.2 | 405.2 | -85 | -10 |
| isoDCA | 391.3 | 345.31 | -80 | -40 |
| GLCA | 432.3 | 74 | -70 | -72 |
| DHLCA | 373.3 | 373.3 | -60 | -10 |
| isoLCA | 375.3 | 375.3 | -50 | -10 |
| T-B-MCA | 514.3 | 124 | -115.1 | -58 |
| T-a-MCA | 514.3 | 123.9 | -90.1 | -61 |
| 12-OCDCA | 405.3 | 405.3 | -122.3 | -10 |
| TCDCA | 498.3 | 123.9 | -90.2 | -60 |
| 7-ketoLCA | 389.3 | 389.3 | -130 | -10 |
| TCA | 514.3 | 124.1 | -114 | -61 |
| DHCA  LCA | 401.2  375.3 | 401.2  375.32 | -86  -70 | -10  -10 |
| HDCA | 391.3 | 391.31 | -130 | -10 |
| CA | 407.3 | 343.2 | -40 | -40 |
| GCA | 464.3 | 402.3 | -86 | -42 |
| GDCA | 448.3 | 73.91 | -90 | -77 |
| TUDCA | 498.3 | 124.01 | -84 | -59 |
| GUDCA | 448.3 | 386.5 | -66.4 | -43 |

Continued table

| DCA | 391.3 | 345.2 | -65 | -40 |
| --- | --- | --- | --- | --- |
| GCDCA | 448.3 | 73.9 | -42 | -84 |
| ACA | 407.3 | 361.2 | -65 | -43 |
| GHDCA | 448.3 | 74.1 | -86 | -84 |
| CDCA | 391.31 | 391.31 | -130 | -10 |
| UDCA | 391.3 | 391.3 | -130 | -10 |
| AILCA | 375.3 | 375.31 | -130 | -10 |
| HCA | 407.3 | 407.31 | -130 | -10 |
| GHCA | 464.3 | 74 | -104.5 | -85 |
| 12-ketoLCA | 389.3 | 389.31 | -96 | -10 |
| UCA | 407.2 | 343.2 | -130 | -42 |
| 3-H-7,12-Dkca | 403.2 | 403.2 | -120 | -10 |
| a-MCA | 407.3 | 407.3 | -51 | -10 |
| 6,7-diketoLCA | 403.3 | 403.3 | -10 | -12 |
| CA-d4 | 411.3 | 347.2 | -130 | -48 |
| GCA-d5 | 469.3 | 407.3 | -80 | -43 |
